# Reliability and validity evaluation of the chinese revision of the attitude towards adult vaccination scale

**DOI:** 10.1186/s12889-023-15684-x

**Published:** 2023-05-12

**Authors:** Jie Kong, Chunguang Liang, Dongmei Fu, Liying Wang, Xiangru Yan, Sisi Li, Hui Zhang

**Affiliations:** 1 School of Nursing, Jinzhou Medical University, No 40, Section 3, Songpo Road, Jinzhou, 121001 China; 2 School of Medicine, Panjin Vocational and Technical College, Panjin, China; 3 School of Medicine, Wuhan College of Arts and Science, Wuhan, China

**Keywords:** Vaccination, Attitude scale, Chinese adults, Adult vaccination attitudes, Psychometric properties

## Abstract

**Background:**

Although vaccination is one of the critical interventions to address global health issues, inadequate vaccination rates has become an international challenge. Vaccine hesitancy is the key to affecting inadequate vaccination rates. According to the WHO SAGE working group’s definition, vaccine hesitancy refers to delaying or refusing vaccination and has been ranked as one of the top 10 health threats. There has yet to be a scale that evaluates vaccination attitudes among Chinese adults. However, an attitude quantity, the adult vaccination attitude scale, has been developed to assess adult vaccination attitudes and reasons for vaccine hesitancy.

**Objective:**

The Adult Attitudes to Vaccination Scale (ATAVAC) was initially developed by Professor Zoi Tsimtsiou et al. This study aimed to analyze the structure of the Chinese version of the ATAVAC and explore the relationship between adult vaccination attitudes, e-health literacy, and medical distrust.

**Methods:**

After obtaining author permission for the initial scales, the study was translated using the Brislin back-translation method. 693 adults were enrolled to the study. To validate this hypothesis, participants finished the socio-demographic questionnaire, the Chinese version of the ATAVAC, the electronic Health Literacy Scale (e-HEALS) and the Medical Mistrust Index (MMI). The exploratory factor analysis (EFA) and confirmatory factor analysis (CFA) were used to examine the underlying structure of the factors of the Chinese version of the Adult Vaccination Attitude Scale and to measure its reliability and validity.

**Results:**

The Cronbach’s alpha coefficient for the Chinese version of the ATAVAC was 0.885, with Cronbach’s alpha coefficients ranging from 0.850 to 0.958 for each dimension. The content validity index was 0.90, and the retest reliability was 0.943. The exploratory factor analysis (EFA) supported the 3-factor structure of the translation instrument, and the scale had good discriminant validity. The confirmatory factor analysis (CFA) revealed a degree of freedom of 1.219, a model fit index (GFI) of 0.979, a normative fit index (NFI) of 0.991, a Tucker-Lewis index (TLI) of 0.998, a comparability index (CFI) of 0.998 and a root mean square error of approximation (RMSEA) of 0.026.

**Conclusion:**

The results show that the Chinese version of the ATAVAC has demonstrated good reliability and validity. Hence, it can be used as an effective tool to assess vaccination attitudes among Chinese adults.

## Introduction

Vaccination is widely recognized worldwide as the most successful and cost-effective intervention to reduce the burden of infectious diseases and as an effective means to reduce the incidence of chronic infectious diseases [1, 2]. However, the World Health Organization’s (WHO) surveillance data show that vaccine hesitancy is widespread in many countries [3, 4]. Chinese also delay or refuse vaccination because of negative information about vaccination risks [5]. Vaccine hesitation reduced the vaccination rate, causing the lack of individual and herd immunity, leading to the rebound of infectious diseases [6]. In 2019, the WHO named it one of the top 10 health threats [7]. Therefore, it is essential to analyze the reasons for vaccine hesitancy and take active measures against it.

Vaccine hesitancy is a long-standing barrier to controlling infectious disease epidemics and faces the serious public health consequences of vaccine-preventable disease [8]. Global studies on the vaccination rate of human papillomavirus (HPV) have shown that the vaccination rate has dropped from 70% to 0.6% due to the misunderstanding of adverse reactions to the HPV vaccine [9]. Misinformation about measles, mumps and rubella vaccines has significantly reduced the coverage of such vaccines in Sweden [10]. In addition, the rising anti-vaccination campaign has caused rising vaccine hesitancy in many countries [11].

Similarly, vaccine hesitancy is widespread and becoming a growing focus in China [12]. The survey showed that China’s influenza vaccine coverage rate is estimated to be 1.5%–2.2% [13]. In China, HPV is a self-paid vaccine with a coverage range of 3.3%–14.09% [14], much lower than that in developed and other developing countries [15, 16]. During the COVID-19 pandemic, despite the significant effect of the COVID-19 vaccine in preventing the severe consequences of COVID-19, the Chinese are becoming increasingly hesitant about vaccination, as observed by Wang et al. [17]. Another study showed that when China first started receiving the COVID-19 vaccine, most respondents were willing to get vaccinated, but their willingness declined sharply within two months [18].

The current study found vaccine hesitancy associated with the following factor: Vaccine hesitancy occurs when awareness of the necessity of vaccination is low (called complacency), concern about its efficacy and safety (called low confidence), and lack of vaccine availability (called convenience) [19]. Despite the relatively high level of health literacy among [20], they are suspected to be the most hesitant group to receive [21]. Since China is the most populous country globally and is increasingly associated with other countries, addressing vaccine hesitancy is crucial to increasing vaccine coverage among Chinese adults. However, no evaluation tool is specifically for adult vaccination attitudes in China. To assess adult vaccination attitudes and further explore the reasons for vaccine hesitancy, Zoi Tsimtsiou et al. developed the scale for adult vaccination attitudes (ATAVAC) [22]. ATAVAC is a concise and practical tool for evaluating general perceptions and attitudes towards adult vaccination.

This study hypothesizes that there would be some changes in the structure and items of ATAVAC in the Chinese population and that Chinese adult vaccine hesitancy is associated with health literacy and medical distrust. Hence, this study aimed to translate the original ATAVAC into Chinese to test its validity and reliability among Chinese adults and to explore the correlation between adult vaccination attitudes and e-health literacy and medical mistrust index.

## Methods

### Research design and participants

The research was a cross-sectional study conducted in China from October to December 2022. The data was obtained through China’s online data collection platform “Questionnaire Star”. A total of 727 people participated in the survey, and after excluding invalid questionnaires, 693 were returned, giving a valid return rate of 95.32% for the questionnaire. The survey was anonymous, and only 60 candidates were requested to leave their personal communication information to assess the reliability of the retest after three weeks. The participants were all native Mandarin speakers and provided informed consent before participating in this study. The inclusion criteria were as follows. (a) aged ≥ 18 years, (b) without communication impairment (deaf or blind), (c) who provided informed consent and volunteered to participate in this study.

### Instruments

#### Questionnaire on general demographic characteristics

A questionnaire on demographic characteristics was designed according to the study’s objectives and concerning relevant literature. It focuses on age, gender, education level and occupation.

#### Attitudes to adult vaccination (ATAVAC) scale

The Adult Attitudes to Vaccination Scale (ATAVAC) was initially developed by Professor Zoi Tsimtsiou et al. to evaluate attitudes toward adult immunization [22]. The scale consists of 11 main items and three dimensions: perceived barriers, safety issues, and the value of adult vaccination. The scale was scored using a 6-point Likert scale ranging from 1 (strongly disagree) to 6 (strongly agree). The total score was calculated by summing the scores of 11 items (reversing 4 reverse items) and dividing them by 11, and higher values indicated better attitudes towards adult vaccination.

#### Chinese version of the Medical Mistrust Index (MMI)

The c-MMI is a commonly used scale to assess patient distrust in healthcare settings and has been applied in multiple populations [23]. There are 17 items, all on a 4-point Likert scale, with scores ranging from 1 (strongly disagree) to 4 (strongly agree), with higher scores indicating higher levels of mistrust.

#### e-health literacy scale (e-HEALS)

The scale is used to assess the ability to search for, understand, and evaluate health information on electronic resources, as well as the ability to use the obtained information available to process and solve health problems. Norman and Skinner initially developed the scale in 2006. It contained eight items, all on a five-point Likert scale, with scores ranging from 1 (very inconsistent) to 5 (very consistent), with higher scores representing higher self-perceived e-health literacy. The Cronbach alpha coefficient is 0.826 [24].

### Procedures

#### Translation and cultural adaptation

The scales were translated into a Chinese version with cultural adaptation after obtaining permission from Professor Zoi Tsimtsiou. A forward-backward translation approach was used based on Brislin’s translation method [25]. Firstly, the ATAVAC was translated into Chinese independently by two MA students majoring in English. Secondly, these two students and the researchers got a draft Chinese version of the questionnaire by comparing the translated Chinese version and discussing and correcting any inconsistencies. A medical expert and a psychologist then back-translated the translations without looking at the original scales. Finally, three nursing professors were invited to discuss and compare the original scale, the draft Chinese translation and the back-translation of the English scale. Controversial items were modified to reinforce language and cultural adaptations to make the scale more appropriate for China.

A pre-survey of 20 adults was selected to verify that the items of the translated scale were readable and comprehensible. The results showed that the scale was readily comprehensible and convenient to fill out (about 5 min to fill out), so a Chinese version of the ATAVAC scale was developed.

## Data Analysis

### Reliability analysis

Version SPSS 25.0 (IBM SPSS Statistics 25.0, Armonk, NY, USA) and version AMOS 26.0 (SPSS, Chicago, IL, USA) serve as tools for data analysis. The Cronbach alpha coefficient corrects for aggregate correlation and retest dependability to measure the internal consistency of the scale. The Cronbach alpha A coefficient equal to or > 0.70 is deemed to be acceptable [26]. The standard value of the corrected item-total correlation was 0.3 [27]. The stability of the scale is determined by the retest correlation coefficient, also known as the retest reliability coefficient, to evaluate the stability of the scale.

### Validity analysis

#### Content validity

It was assessed by seven experts using the Delphi expert consultation method. Content validity was indexed by (CVI), including item-level content validity index (I-CVI) and mean S-CVI (S-CVI / Ave) [28]. These seven experts scored the relevance of all items and the corresponding dimensions. CVI was calculated using a 4-point scale (1 = no correlation, 2 = low correlation, 3 = strong correlation, 4 = very strong correlation).

#### Discriminant Validity

In the discriminant validity analysis, the scales were divided into two groups based on their aggregate scores: the top 27% were the high group, and the bottom 27% were the low group, and item scores in both groups were analyzed using a two-tailed independent samples t-test. Discriminant validity was good when item scores for both groups reached a significant level (*p* < 0.05).

#### Construct validity

The construct validity of the Chinese version of the Adult Vaccination Attitude Scale was examined using both exploratory factor analysis (EFA) and validation factor analysis (CFA). First, we classified the entire data randomly into two parts. One part comprised 355 participants for the EFA, and the other comprised 338 participants for the CFA.

The Kaiser-Meyer-Olkin (KMO) [29] metric and Bartlett’s sphericity test [30] were applied to evaluate the ability of factor the correlation matrix in Sample 1 (n = 355) used for EFA. The measure is applicable for factor analysis only when the KMO > 0.6 and Bartlett’s sphericity test is statistically significant (*p* < 0.05).

Within Sample 2 (n = 338), a CFA was carried out to validate the consistency of the model structure with the explored factor structure. CFA can facilitate the further assessment of the consistency of the model with the factor structure [31] CMIN/DF, Comparative Fit Index (CFI), Goodness of Fit Index (GFI) and Tucker-Lewis Index (TLI) were used to indicate the fit of the model. The nearer the CMIN/DF value is to 0, the better the fit of the model [32, 33]. When the values of CFI, GFI and TLI are ≥ 0.9, it means that the model fits well [32, 34]. RMSEA is used to evaluate the degree of mismatch of the model, and the closer its value is to 0, the better the model fit is [32, 34].

### Ethical approval

Each participant completed an informed consent form. The information in each questionnaire was protected. Moreover, This study was approved by the Ethics Committee of the Jinzhou Medical University (JZMULL2021009) and the process followed the code of ethics provided by the Ethics Committee.

## Results

### Demographics and sample characteristics

693 participants met the criteria for inclusion: 545 females (78.6%) and 148 males (21.4%). Respondents were distributed in descending order of age, with the highest proportion in the 18–30 age group (52.5%). Most participants were office bearers (56.4%) and students (27.0%). The overall education level of the participants was high, with the highest proportion (73.9%) being college or bachelor’s degree holders. Providing further details on the demographics are shown in Table 1. Table 2 shows the participants’ mean (SD) scores on each item in the ATAVAC Chinese Revision.

**Table 1:** Demographic characteristics

Variable		Total (N%)
Age (years old) Gender Educational level	18–29 30–39 40–49 ≥ 50 Male Female Junior high school and below High school or technical secondary school Junior College or undergraduate Postgraduate and above	364(52.5) 106(15.3) 108(15.6) 115(16.6) 148(21.4) 545(78.6) 71(10.2) 98(14.1) 512(73.9) 12(1.7)
Profession	Work Retire Unemployment Students	391(56.4) 82(11.8) 33(4.8) 187(27.0)

**Table 2:** Mean (SD) scores for all participants in the Chinese Revised Adult Vaccination Attitude Scale(*N* = 693)

Items on the Chinese Revised Adult Vaccination Attitude Scale	Mean (SD)
1 I fear the immediate complications of a vaccine (such as allergic reactions).	4.47(1.023)
2 I fear the potential impact of vaccines on my health in the future.	4.55(1.000)
3 It is difficult for me to access the doctor for vaccination (I cannot find an appointment or the office is too far away or there is no transportation, etc).	4.57(1.024)
4 It is difficult for me to access the doctor for vaccination (I cannot find an appointment or the office is too far away or there is no transportation, etc).	4.78(1.039)
5 I believe in the value of vaccination.	4.42(0.710)
6 I believe that vaccines are necessary for adults.	4.42(0.716)
7 I believe that the benefits of vaccination outweigh the potential risks.	4.37(0.751)
8 I think if I get ill, I will get more antibodies (better body auto-defense) than if I just get a vaccination.	3.38(1.657)
9 I believe that vaccines are very effective in protecting me from getting a disease.	4.23(0.797)
10 I haven’t had a vaccine as an adult so far, so I don’t need it.	4.76(0.987)
11 I believe that vaccines should only be given to children.	4.86(0.957)

### Item Analyze

The scale’s items (11 items) were analyzed. The reliability analysis revealed an overall Cronbach’s alpha coefficient of 0.885. However, the scale’s internal consistency would have been enhanced by removing item 8, as detailed in Table 3. the revised item 8 (correlation < 0.3) had a low correlation with the overall score. The I-CVI for item 8, assessed by experts for content validity, was 0.429. therefore, item 8 was removed after the expert comment.

**Table 3:** Cronbach alpha if the item is deleted (N = 693)

Itme	Cronbach’s Alpha if Item Deleted	Corrected Item-Total Correlation
1 2 3 4 5 6 7 8 9 10 11	0.810 0.805 0.809 0.808 0.802 0.801 0.801 **0.885** 0.808 0.811 0.810	0.546 0.603 0.551 0.561 0.720 0.731 0.713 **0.105** 0.601 0.533 0.546

### Reliability analysis

Reliability analysis can reflect the reliability and stability of the scale measurement results, and the better the reliability is, the more reliable the measurement results are. The overall Cronbach’s alpha coefficient for the scale was 0.885. the Cronbach’s alpha coefficients for each dimension ranged from 0.850 to 0.958. the retest reliability obtained from a random sample of 60 people after 3 weeks was 0.943, indicating that the scale is less subject to time interference and has good stability. It can be concluded that the revised Chinese Adult Vaccination Attitude Scale has appropriate reliability. (Table 4)

**Table 4:** Reliability analysis for Chinese version of the ATAVAC

The scale and its dimension	Cronbach^’^s Alpha	Test-retest reliability
The ATAVAC Safety issues Adult vaccination value Perceived barriers	0.885 0.958 0.920 0.850	0.943

### Validity analysis

#### Content validity

Seven experts evaluated ATAVAC Chinese content validity. The expert panel consisted of three psychologists and four skilled medical experts from China and the UK. Each item was scored by each expert separately. The results of the content validity analysis showed that item 8 (I get more antibodies than vaccination) had a low I-CVI of 0.429, indicating that item 8 is not suitable for the Chinese population, which is consistent with the results of our statistical analysis. The remaining items ranged from 0.857 to 1.000, and the S-CVI was 0.900.

#### Discriminant Validity

The total score of the Chinese version of ATAVAC is arranged in descending order. Points ranked in the top 27% were divided into one group and in the final 27% were divided into another group. Two independent samples t-tests were used to analyze the differences between the two groups. The results were statistically significant (*p* < 0.05); this study showed that the cut-off scores were 4.2 and 5.0, and the results showed that both the high and low scores were statistically significant (*p* < 0.05) and had good discriminant validity to assess the level of response of the various participants effectively. The results are shown in Table 5.

**Table 5:** score comparison between high-score and low-score groups (N = 693)

Item	Low-score group (n = 221), Mean (SD)	High-score group (n = 200), Mean (SD)	t-test(df)	*p*-value
1 2 3 4 5 6 7 9 10 11	3.57(0.872) 3.61(0.861) 3.74(0.927) 3.89(1.020) 3.36(0.648) 3.65(0.651) 3.56(0.650) 3.48(0.652) 3.97(1.015) 4.09(1.042)	5.23(0.670) 5.31(0.607) 5.30(0.728) 5.49(0.561) 4.99(0.095) 5.00(0.067) 4.97(0.189) 4.89(0.417) 5.45(0.534) 5.46(0.591)	-21.600 -22.968 -18.975 -19.509 -28.434 -28.831 -29.348 -25.890 -18.267 -16.245	＜0.001 ＜0.001 ＜0.001 ＜0.001 ＜0.001 ＜0.001 ＜0.001 ＜0.001 ＜0.001 ＜0.001

### Construct validity

#### Exploratory factor analysis (EFA)

**Fig. 1:**
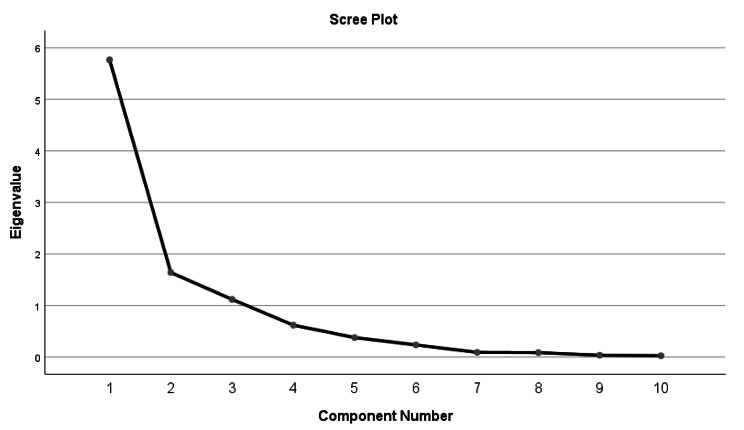
Screen plot of exploratory factor analysis for the Chinese version of the ATAVAC(n = 355)

The decomposability of the sample (n = 355) matrix was tested before starting the EFA. In this study, The χ ^2^ value of Bartlett’s spherical test is 4297.841 (*p* < 0.001), and the KMO value is 0.803. KMO greater than 0.5 is suitable for factor analysis. After applying the data to PCA with an orthogonal rotation of the maximum variance, three factors with feature root greater than 1 were extracted, the number of factors is the same as the original scale. The cumulative variance contribution rate was 65.235%, and the load value of each item was > 0.4. The load matrix of each factor is shown in Table 6. The gravel plot further explains the structure of the 3 factors, with a weaker downward trend after point 3. The gravel diagram is shown in Fig. 1.

**Table 6:** Factor loadings of the exploratory factor analysis with 10 items (n = 355)

Item	Factor1	Factor2	Factor3
1	0.891		
2	0.909		
3			0.850
4			0.834
5		0.897	
6		0.911	
7		0.895	
9		0.861	
10		0.903	
11		0.879	

#### Confirmatory factor analysis (CFA)

**Fig. 2:**
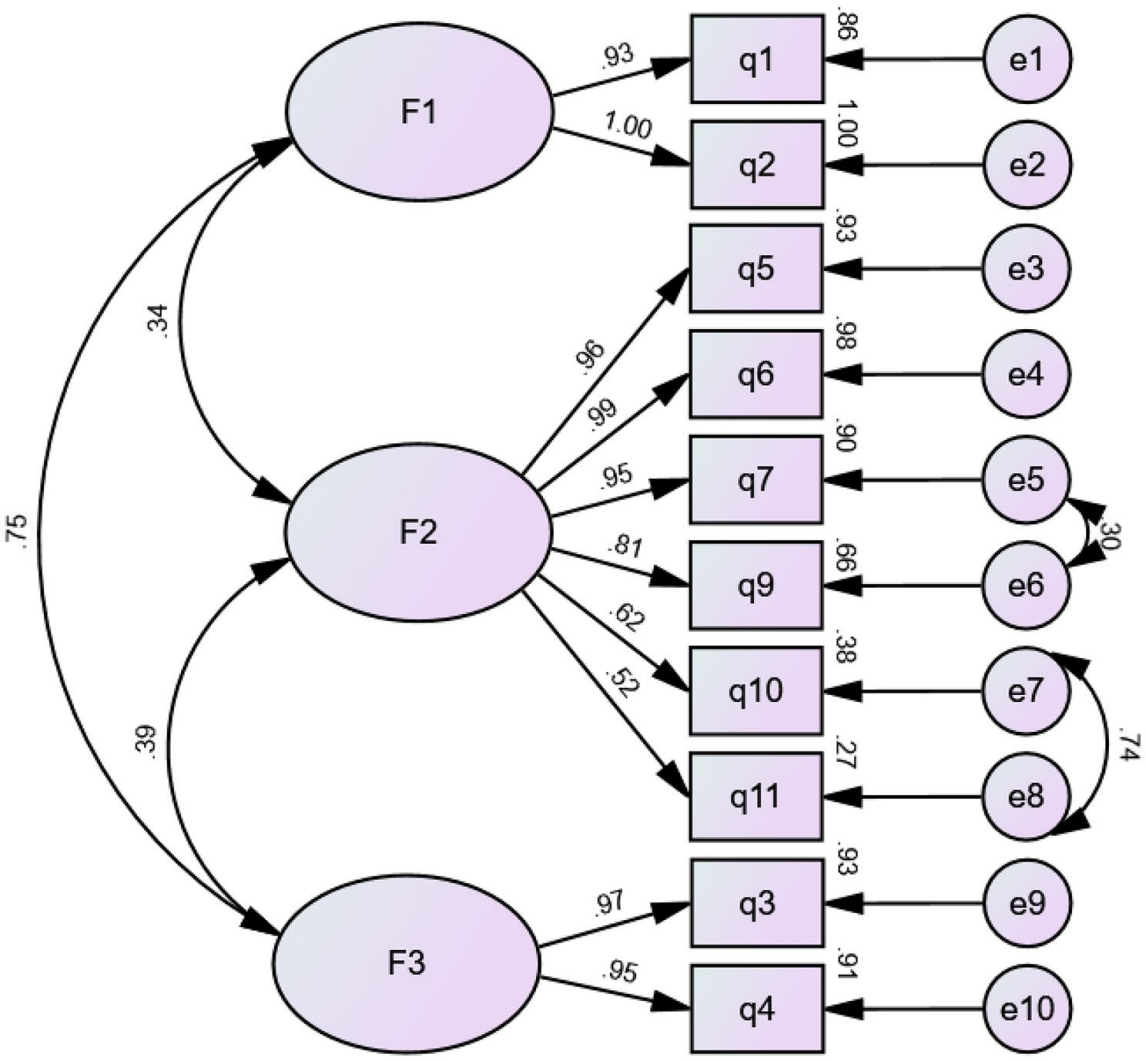
Standardized three-factor structural model of the Chinese version of the ATAVAC(n = 338). F1(Safety issues, two items), F2(Adult vaccination value, six items), F3(Perceived barriers, two items)

The purpose of the confirmatory factor analysis is to verify whether the relationship between the item and the factor is consistent with the hypothesis. CFA analysis was performed for the sample 2 (n = 338). In this study, the validation results showed that the accessories had good results. The values of these indicators are given as followsχ^2^ /df = 1.219, CFI = 0.998, GFI = 0.979, NFI = 0.991, TLI = 0.998, RMSEA = 0.026. The CFA results are shown in Fig. 2.

### Relativity

Table 7 shows the factors associated with ATAVAC scores in China: China ATAVAC score is positively correlated with e-health literacy and negatively correlated with medical distrust index.

**Table 7:** Pearson’s correlations between the ATAVAC Correlations count and e-health literacy and medical distrust index

	1	2	3
1 ATAVAC	--	--	--
2 Electronic health literacy	0.730^**^	--	− 0.434^**^
3 Medical distrust index	− 0.499^**^	--	--

ATAVAC: The Attitude Towards Adult Vaccination scale; MMI: Chinese version of the Medical Mistrust Index; e-HEALS: eHealth Literacy scale; ** *p* < 0.01; * *p* < 0.05

## Discussion

Vaccine hesitancy is a crucial factor contributing to decreased vaccination coverage and the resumption of infectious disease [35]. There are indeed scales that assess vaccine hesitancy. However, they are limited to children or high-income groups, such as the Parent Attitudes towards Child Vaccination Scale (PACV) developed by Opel et al. [36, 37]. SAGE constructed the vaccine hesitancy scale (VHS) based on the determinant matrix and a previously validated [38]. The KATE-S scale also assesses parental vaccine [39]. Meanwhile, adult vaccination is crucial in achieving herd immunity [40, 41]. Therefore, it is urgent to identify the causes of adult vaccine hesitancy and to propose solutions. The ATAVACA are scale specially developed for adults to assess specific perceptions and feelings of adult vaccination and related barriers. Moreover, the application of the ATAVAC scale helps to play an essential role in addressing low adult vaccination rates and improving adult vaccination motivation.

This study shows that a Chinese version of the attitude towards adult vaccination scale (ATAVAC) has a three-factor model that explains 65.235% of the total variance and has good psychometric characteristics. The ATAVAC has good internal consistency, test-retest reliability, construct validity,content validity and discriminant validity. Lastly, a Chinese scale comprising 10 items and a 3-factor structure was developed.

### The chinese version of ATAVAC has excellent reliability

Reliability analysis reflects the stability of the scale’s structure [42]. The scale’s reliability was used to evaluate Cronbach’s alpha coefficient, item-total correlation and test-retest. In our study, Cronbach’s alpha coefficient for the Chinese version of the ATAVAC was 0.885, indicating that the ATAVAC is sufficiently stable for measuring attitudes toward adult vaccination. The item-score correlation coefficients were all higher than 0.30 (except for item 8), indicating good internal consistency of the Chinese version of the ATAVAC. In addition, the test-retest reliability of the Chinese version of the ATAVAC was also good, indicating that the scale has good stability over time. The results showed that the Chinese version of the ATAVAC has excellent reliability.

### The chinese version of the ATAVAC has excellent validity

Effectiveness refers to the degree to which the instrument being tested corresponds precisely to the world’s reality [43]. The scale’s validity is evaluated using discriminant, construct, and content validity. The discriminant validity results of the Chinese version of the ATAVAC revealed that all items in the 2 groups scored at a significant level (*p* < 0.05) and were considered good. The I-CVI and S-CVI of the ATAVAC were higher than the reference values [44] and had appropriate content reliability. This study extracted three factors by exploratory factor analysis, explaining 65.235% of the total data variance. Factor loadings for the 10 items ranged from 0.850 to 0.958. In addition, the CFA showed that the model fit indicators all met acceptable standards, making the scale well-suited to a structural model of the three dimensions.

### There is a plausible explanation for removing an item

The original scale constructed a three-factor structural model consisting of 11 items. Factor 1-safety issues (including items 1,2), Factor 2-adult vaccination value (including items 5,6,7,8,9,10,11), Factor 3-perceived barriers (including items 3,4). The Chinese version of the ATAVAC supports a three-factor structural model consisting of 10 items. Factor 1-safety issues (including items 1,2), Factor 2-adult vaccination value (including items 5,6,7,9,10,11), Factor 3-perceived barriers (including items 3,4). In our study, the number of dimensions and factor attribution was the same as in the original questionnaire, but the number of items is slightly different.

On the one hand, it is related to domestic cultural backgrounds and foreign countries. To ensure the accuracy of the semantics of the scale items and their intelligibility in the target population, the items were adjusted during translation, which may have affected the initial structure of the scale. On the other hand, it is related to differences in vaccination policies and public prevention strategies. Countries address the conflict between vaccination obligations and refusal in different ways: some force vaccination, while others promote vaccination [45]. Furthermore, our study is a study related to “attitude.“ Attitude is a psychological tendency with an intense subjectivity. Allport once pointed out that attitude is the most crucial concept in contemporary American social psychology, which determines a person’s thoughts and behavior [46]. Public attitude towards vaccination is critical to improved vaccination rates to achieve herd immunity, especially for novel infectious diseases [47]. Meanwhile, the experimental results showed a significant increase in Cronbach’s α coefficient when removing item 8 and the content validity was assessed by the experts. The I-CVI of item 8 was 0.429. Therefore, the expert group decided to delete item 8.

### Correlation of adult vaccination attitudes with e-health literacy (e-HEALS) and the Medical Mistrust Index (MMI)

In our study, e-health literacy was positively correlated with adult vaccination attitude count score: higher e-health literacy indicates higher motivation for vaccination. E-health literacy refers to the ability of people to find, discover, understand and evaluate health information from electronic resources and apply this knowledge to solve individual health problems or make decisions about health [48]. Residents with high e-health literacy can often use online resources to obtain valuable health data and better apply them to practice. Residents with higher e-health literacy are more proactive [49] in preventing disease-related behaviors. A correct understanding of vaccination knowledge was significantly associated with the vaccination attitude [50–52]. Lack of knowledge is an essential factor in confounding the vaccination effectiveness. Electronic media, like the Internet, are the primary way people obtain vaccine information. Therefore, the government should establish active and healthy online information platforms to encourage the public to obtain vaccine knowledge through official channels, eliminate misunderstandings about vaccines, and increase vaccine confidence.

This study showed a negative correlation between medical mistrust and adult vaccination attitude count scores. Steven Taylor et al. showed that vaccine refusal is closely related to vaccine mistrust [53]. Healthcare providers are vital in influencing public trust in scientific and epidemiological evidence [54]. The willingness to get vaccinated is a matter of trust: vaccines are necessary and safe. However, the recent vaccination-related adverse events and counterfeit vaccine examples represent a significant decline in public trust in healthcare professionals and vaccine developers [55]. Therefore, China should continue to improve the reputation system of the vaccine industry to ensure the quality and safety of vaccines from research and development to circulation. In addition, improving the service level of vaccination medical personnel and cultivating practical communication skills between doctors and patients are also essential to improve residents’ trust in the vaccine and the vaccination rates.

In China, preferential policies and incentives for the cost of vaccination have been implemented to encourage [56]. However, due to the limited health personnel and vaccine shortage, the waiting time for vaccination is often very long, which causes great inconvenience to the vaccinees and may lead to their hesitancy [57–59]. Therefore, in addition to improving public health literacy and trust in medical care, but also should consider the key measures include: simplifying the vaccination procedures and improving the convenience of vaccination, reasonable distribution of vaccination clinics and personnel.

## Limitations

First, the large proportion of young and highly educated women in our sample may limit the generalizability to other populations. Moreover, it should be validated with broader adult populations in the future. Second, this study’s data are the participants’ self-assessed outcomes, and bias is inevitable. Therefore, the reliability and validity of this scale should be analyzed and validated in more depth in future studies.

## Conclusion

This Chinese version of ATAVAC comprises 10 items, supporting the three-factor structure and showing excellent validity and reliability. After cultural adjustment, the scale is simple and easy to understand, which is more suitable for Chinese people. Furthermore, the scale is used to assess adult attitudes towards vaccination which is beneficial for analyzing the reasons for low adult vaccination rates and providing effective interventions for developing active vaccine policies and reducing the prevalence of certain adult infectious diseases. The scale also has clinical value for its application in terms of “vaccine hesitancy” and the smooth solution of public health problems.

## Data Availability

The datasets generated and/or analysed during the current study are not publicly available due Chinese people are relatively secretive about their lives and thoughts, although informed consent was obtained from study subjects prior to the survey and the findings were largely reported but are available from the corresponding author on reasonable request.“

## Abbreviations

ATAVAC: The Attitude Towards Adult Vaccination scale
MMI: Chinese version of the Medical Mistrust Index
e-HEALS: eHealth Literacy Scale
PCA: Principal components analysis
EFA: Exploratory Factor Analysis
CFA: Confirmatory Factor Analysis
CFI: Comparative Fit Index
LI: Tucker-Lewis Index
RMSEA: Root Mean Square Error of Approximation
GFI: goodness-of-ft index
NFI: normed fit index
HBV: Hepatitis B Virus
